# Multi-modal Brain MRI in Subjects with PD and iRBD

**DOI:** 10.3389/fnins.2017.00709

**Published:** 2017-12-19

**Authors:** Silvia Mangia, Alena Svatkova, Daniele Mascali, Mikko J. Nissi, Philip C. Burton, Petr Bednarik, Edward J. Auerbach, Federico Giove, Lynn E. Eberly, Michael J. Howell, Igor Nestrasil, Paul J. Tuite, Shalom Michaeli

**Affiliations:** ^1^Department of Radiology, Center for Magnetic Resonance Research (CMRR), University of Minnesota, Minneapolis, MN, United States; ^2^Department of Pediatrics, University of Minnesota, Minneapolis, MN, United States; ^3^Central European Institute of Technology (CEITEC), Masaryk University, Brno, Czechia; ^4^MARBILab, Centro Fermi - Museo Storico Della Fisica e Centro di Studi e Ricerche Enrico Fermi, Rome, Italy; ^5^Department of Applied Physics, University of Eastern Finland, Kuopio, Finland; ^6^Fondazione Santa Lucia IRCCS, Rome, Italy; ^7^Division of Biostatistics, University of Minnesota, Minneapolis, MN, United States; ^8^Department of Neurology, University of Minnesota, Minneapolis, MN, United States

**Keywords:** rotating frame MRI, Parkinson's disease, iRBD, functional connectivity, DTI

## Abstract

Idiopathic rapid eye movement sleep behavior disorder (iRBD) is a condition that often evolves into Parkinson's disease (PD). Therefore, by monitoring iRBD it is possible to track the neurodegeneration of individuals who may progress to PD. Here we aimed at piloting the characterization of brain tissue properties in mid-brain subcortical regions of 10 healthy subjects, 8 iRBD, and 9 early-diagnosed PD. We used a battery of magnetic resonance imaging (MRI) contrasts at 3 T, including adiabatic and non-adiabatic rotating frame techniques developed by our group, along with diffusion tensor imaging (DTI) and resting-state fMRI. Adiabatic T_1ρ_ and T_2ρ_, and non-adiabatic RAFF4 (Relaxation Along a Fictitious Field in the rotating frame of rank 4) were found to have lower coefficient of variations and higher sensitivity to detect group differences as compared to DTI parameters such as fractional anisotropy and mean diffusivity. Significantly longer T_1ρ_ were observed in the amygdala of PD subjects vs. controls, along with a trend of lower functional connectivity as measured by regional homogeneity, thereby supporting the notion that amygdalar dysfunction occurs in PD. Significant abnormalities in reward networks occurred in iRBD subjects, who manifested lower network strength of the accumbens. In agreement with previous studies, significantly longer T_1ρ_ occurred in the substantia nigra compacta of PD vs. controls, indicative of neuronal degeneration, while regional homogeneity was lower in the substantia nigra reticulata. Finally, other trend-level findings were observed, i.e., lower RAFF4 and T_2ρ_ in the midbrain of iRBD subjects vs. controls, possibly indicating changes in non-motor features as opposed to motor function in the iRBD group. We conclude that rotating frame relaxation methods along with functional connectivity measures are valuable to characterize iRBD and PD subjects, and with proper validation in larger cohorts may provide pathological signatures of iRBD and PD.

## Introduction

Idiopathic rapid eye movement (REM) sleep behavior disorder (iRBD) is a sleep disorder associated with abnormal pontomedullary synuclein protein. iRBD evolves into Parkinson's disease (PD) or another synucleinopathy, e.g., dementia with Lewy bodies (LBD) or multiple system atrophy (MSA), in up to 90% of cases (Schenck et al., 1986, 1996; Boeve et al., 2001; Luk et al., 2012; Boeve, 2013; Bunzeck et al., 2013). Thus, iRBD may provide an opportunity to understand the evolution of neurodegeneration as well as a window in which to intervene with disease altering therapies. It is thought that the α-synuclein pathology in more caudal brainstem regions in iRBD ascends to the substantia nigra (SN) of the midbrain as the disease evolves into PD or other synuclein disorders (Boeve, 2013). Iron, which is present in higher quantities in the substantia nigra and other structures, may also play a role in the loss of dopaminergic neurons through its pro-oxidant effects (Zecca et al., 2008; Kaur et al., 2009). In addition, several studies have demonstrated abnormalities in structural integrity and functional connectivity in patients with iRBD (Hanyu et al., 2012; Ellmore et al., 2013).

Magnetic resonance imaging (MRI) offers several non-invasive contrasts that are useful in evaluating the pathological hallmarks of patients with iRBD, and any MRI findings in iRBD patients can be compared with those in individuals with PD. Previous studies have focused on evaluating structural approaches such as diffusion tensor imaging (DTI) and voxel-based morphometry (VBM) in patients with PD. This has included work that showed changes in DTI fractional anisotropy (FA) (Menke et al., 2009, 2010; Vaillancourt et al., 2009; Peran et al., 2010; Du et al., 2011) and mean diffusivity (MD) (Kamagata et al., 2012) in the substantia nigra of patients with PD. Others have shown that DTI may be useful in differentiating PD from other degenerative parkinsonian disorders and may also have the potential to monitor for disease progression (Prodoehl et al., 2013). Additionally, VBM demonstrated non-dopaminergic brainstem alterations in iRBD (Scherfler et al., 2011). VBM and DTI have also provided insights into non-motor features such as depression (Feldmann et al., 2008; Kostic et al., 2010), smell (Ibarretxe-Bilbao et al., 2010; Rolheiser et al., 2011) and cognitive changes (Ibarretxe-Bilbao et al., 2009; Song et al., 2011) in PD. Therefore, DTI and VBM may prove useful in evaluating iRBD as it evolves into a Parkinsonian condition. So far, it has only been possible to show discriminative properties of the MRI methods using fairly large numbers of patients and controls, and these methods have not yet revealed if they are able to characterize the clinical severity of PD (Gorell et al., 1995; Martin et al., 2008). Therefore, our group previously employed novel microstructural imaging methods to map the SN (Michaeli et al., 2007; Nestrasil et al., 2010), as well as other brainstem and subcortical regions, in PD (Karagulle Kendi et al., 2008; Tuite et al., 2012).

In our current work, we employed several MRI modalities that are sensitive to various brain tissue properties, such as microstructural integrity, iron loads, and functional connectivity. Namely, we utilized novel rotating frame relaxation mapping methods developed in our group including adiabatic T_1ρ_ (Michaeli et al., 2006), T_2ρ_ (Michaeli et al., 2004) and RAFF4 (Relaxation Along a Fictitious Field in the rotating frame of rank 4) (Liimatainen et al., 2010, 2011, 2015), together with DTI and resting-state functional MRI (rsfMRI). Our previous studies conducted in PD (Michaeli, 2007; Michaeli et al., 2007; Nestrasil et al., 2010; Tuite et al., 2012) and other brain conditions (Sierra et al., 2008; Jokivarsi et al., 2009; Mangia et al., 2013; Satzer et al., 2015) have shown that the proposed rotating frame methods provide information on different relaxation mechanisms in the human brain. T_2ρ_ is sensitive to diffusion and exchange of water protons in environments with different local magnetic susceptibilities and reflects iron content with higher sensitivity than conventional T_2_ MRI (Michaeli et al., 2005, 2007). T_1ρ_ provides an indication of neuronal loss and can be used to assess PD nigral degeneration with a higher sensitivity than is possible with conventional T_1_-based MRI (Michaeli et al., 2006, 2007). RAFF4 methodology, which is derived from adiabatic T_1ρ_ and T_2ρ_ (Liimatainen et al., 2010, 2011, 2015), has high sensitivity to myelin content (Satzer et al., 2015; Hakkarainen et al., 2016), and thus could provide information on microstructural integrity.

Rotating frame relaxations offer a conceptual advantage as compared to free-precession relaxation methods for characterizing tissue properties thanks to their inherent sensitivity to slower motional regimes (Michaeli et al., 2008). Indeed, while water molecules undergo a large variety of motional regimes in tissue, the motions which are most sensitive to the intricate nature of tissue microstructure and composition are those in intermediate-slow regimes. Rotating frame relaxations during frequency swept pulses offer additional practical advantages as compared to continuous-wave T_1ρ_, including a minimized sensitivity to B_1_ distributions and a capability to simultaneously tune the contrast to multiple effective frequencies (Mangia et al., 2009). Although not common for clinical studies of neurological disorders, rotating frame relaxations with frequency swept pulses were proven as robust and sensitive methods for a variety of *in vivo* investigations conducted by our group (Michaeli et al., 2007; Sierra et al., 2008; Jokivarsi et al., 2009; Nestrasil et al., 2010; Griffith et al., 2011; Liimatainen et al., 2012; Tuite et al., 2012; Mangia et al., 2013; Satzer et al., 2015; Hakkarainen et al., 2016) and others (Andronesi et al., 2014; Casula et al., 2017; Okuaki et al., 2017).

Our previous applications of mapping adiabatic rotating frame relaxations in humans had been limited to single-slice acquisitions performed on a non-clinical 4 Tesla scanner (Michaeli et al., 2007; Nestrasil et al., 2010; Tuite et al., 2012; Mangia et al., 2013). With the present work, we piloted the extension of these methodologies to multi-slice acquisitions on a clinical 3 Tesla scanner, and we thus embarked on a small cross-sectional study of 10 healthy controls, 9 patients with iRBD, and 10 patients with PD. Rather than focusing on the endpoint of progression to a clinical PD diagnosis for iRBD subjects, the ultimate goal is to use 3 T MRI for characterizing the spectrum of abnormalities resulting from inexorable synuclein deposition and other pathological features in both PD and iRBD. Our study is a step forward for establishing biomarkers which can characterize PD and iRBD, and ultimately the development of parkinsonian syndromes from iRBD subjects.

## Methods

### Subjects

PD and iRBD subjects were recruited from the University of Minnesota Movement Disorders Clinic. Ten healthy controls, 9 iRBD patients and 10 individuals with PD were recruited and underwent the MRI protocol. However, all data from one iRBD subject and one PD subject were entirely discarded due to major motion observed during the acquisition. All the other subjects successfully completed the MRI protocol (Table 1), except one control subject who did not finish the DTI acquisitions, and one PD subject who presented excessive motion during the resting-state acquisition.

**Table 1:** Subjects' characteristics.

	**Control mean ± SD (range)**	**iRBD mean ± SD (range)**	**PD mean ± SD (range)**
n	10	8	9
Sex	7M/3F	4M/4F	4M/5F
Age (years)	57.3 ± 5.2 (47.0–63.4)	65.7 ± 6.7 (54.7–72.3)	66.1 ± 6.6 (56.3–74.3)
Disease duration (years)	–	9.1 ± 14.1 (0.1–15.0)	2.4 ± 1.9 (0.1–4.5)
Onset side (PD)	–	–	4L/4R, 1NA
UPDRS	–	11.6 ± 6.2 (3–21)	36.8 ± 11.2 (26–58)
UPDRS-III	–	6.9 ± 4.9 (0–14)	20.3 ± 5.5 (15–32)
MoCA	–	27.3 ± 1.4 (25–29)	27.7 ± 1.7 (25–30)

*Characteristics of subjects whose data underwent further analyses. All subjects completed the whole MRI protocol, except one control subject who did not finish the DTI acquisitions. One PD subject was later excluded from functional connectivity analyses, because the resting-state functional scans exceeded the established criteria for acceptable motion*.

Exclusion criteria included history of stroke, seizures, neurosurgical procedures, arrhythmias, Montreal cognitive assessment (MoCA) scores lower than 25, and incompatibility with MR safety criteria. This study was carried out in accordance with the recommendations of The Code of Federal Regulations, Institutional Review Board, with written informed consent from all subjects. All subjects gave written informed consent in accordance with the Declaration of Helsinki. The protocol was approved by the Institutional Review Board: Human Subjects Committee of the University of Minnesota. Clinical assessments were performed on anti-parkinsonian medications (for subjects on such treatment) and included the National Institute of Neurological Disorders and Stroke (NINDS) Common Data Element PD medical and family history, demographic information, the Hamilton Anxiety and Depression Rating Scales (Hamilton, 1959, 1960, 1980), United States version of the Parkinson disease questionnaire (PDQ-39) that reflects upon quality of life (Bushnell and Martin, 1999), the Unified Parkinson Disease Rating Scale (UPDRS), the Schwab and England, and Hoehn and Yahr (Fahn and Elton, 1987; Goetz et al., 2007, 2008; Dibble et al., 2010). In particular, we also used the UPDRS part III score to characterize our patient populations, as UPDRS part III represents an objective evaluation of motor symptoms. We assessed for features of iRBD with the validated REM sleep behavioral disorder questionnaire-Hong Kong (RBDQ-HK, English version) (Li et al., 2010) in all subjects. For cognitive function, we included the MoCA and the CogState (Nasreddine et al., 2005; Hoops et al., 2009; Gagnon et al., 2010). For smell perception, we used the brief smell identification test (B-SIT) (Hawkes et al., 1997; Double et al., 2003; Bohnen et al., 2008; Silveira-Moriyama et al., 2009).

### Imaging protocol

Studies were performed on a 3 T Siemens Prisma system using a 32-channel RF receive coil. Standard B_0_ shimming were performed at the beginning of the imaging session to minimize B_0_ inhomogeneities.

Adiabatic T_1ρ_, T_2ρ_ and non-adiabatic RAFF4 measurements were collected from 30 AC-PC aligned slices covering the brainstem and basal ganglia using a segmented GRE readout with 4 segments, voxel size = 1.6 × 1.6 × 3.6 mm^3^, GRAPPA = 3, TE = 3.18 ms and TR = 2 s. For adiabatic relaxation measurements, hyperbolic secant (HS) pulses were used with BW = 1.6 kHz, pulse duration Tp = 6 ms, peak power $\omega_{1}^{\text{max}}$ /(2π) = 800 Hz, 5 acquisitions with number of pulses = 0, 4, 8, 12, 16 using MLEV4 phase cycling (Michaeli et al., 2004, 2005, 2006, 2008). In T_2ρ_ acquisitions, magnetization was on -z prior to readout. For RAFF4, T_p_ was 4.52 ms for one P-packet. The number of P-packets was 0, 4, 8, 12, 16 with $\omega_{1}^{\text{max}}$ /(2π) = 327 Hz. RAFF4 acquisitions were separately conducted without and with a global inversion (achieved by the same HS pulse used for T_1ρ_) prior to the train of P-packets, to produce sampling of the recovery and decay curve, respectively, to the steady-state magnetization induced during RAFF4.

T_1_-weighted, T_2_-weighted images, DTI and rsfMRI were collected covering the whole brain. For T_1_-weighted acquisitions MPRAGE with TR = 2150 ms, TE = 2.47 ms, TI = 1,100 ms, voxel size = 1 × 1 × 1 mm^3^, flip angle = 8 degrees and GRAPPA = 2 was used. T_2_-weighted images were collected using SPACE sequence, voxel size = 1 × 1 × 1 mm^3^, TE = 147 ms and GRAPPA = 2. Two DTI datasets were acquired with different phase encoding (i.e., anterior-posterior and posterior-anterior) utilizing TR = 2,820 ms, TE = 72.6 ms; multi band (MB) = 4 (https://www.cmrr.umn.edu/multiband), 7 non-diffusion weighed (b_0_) images, and 93 diffusion weighted images with *b*-value of 750 s/mm^2^ (47 images) and 1,500 s/mm^2^ (46 images) and voxel size = 1.8 × 1.8 × 1.8 mm^3^. Finally, rsfMRI data was obtained using gradient echo Echo Planar Imaging (EPI) sequence, TR = 900 ms, multi band (MB) = 4; TE = 30 ms; voxel size = 3 × 3 × 3 mm^3^, matrix size = 64 × 64, 48 AC-PC aligned slices with interleaved slice acquisition, 502 volumes.

### Image analysis and ROI definition

Brain segmentation of the T_1_-weighted scan was carried out with FreeSurfer (FS) version 5.3.0 (http://surfer.nmr.mgh.harvard.edu) installed on the supercomputer at the Minnesota Supercomputing Institute. Automated processing has been described previously (Fischl et al., 2002). A trained operator visually inspected each subject's data to ensure accuracy of the segmentation.

**Figure 1:**
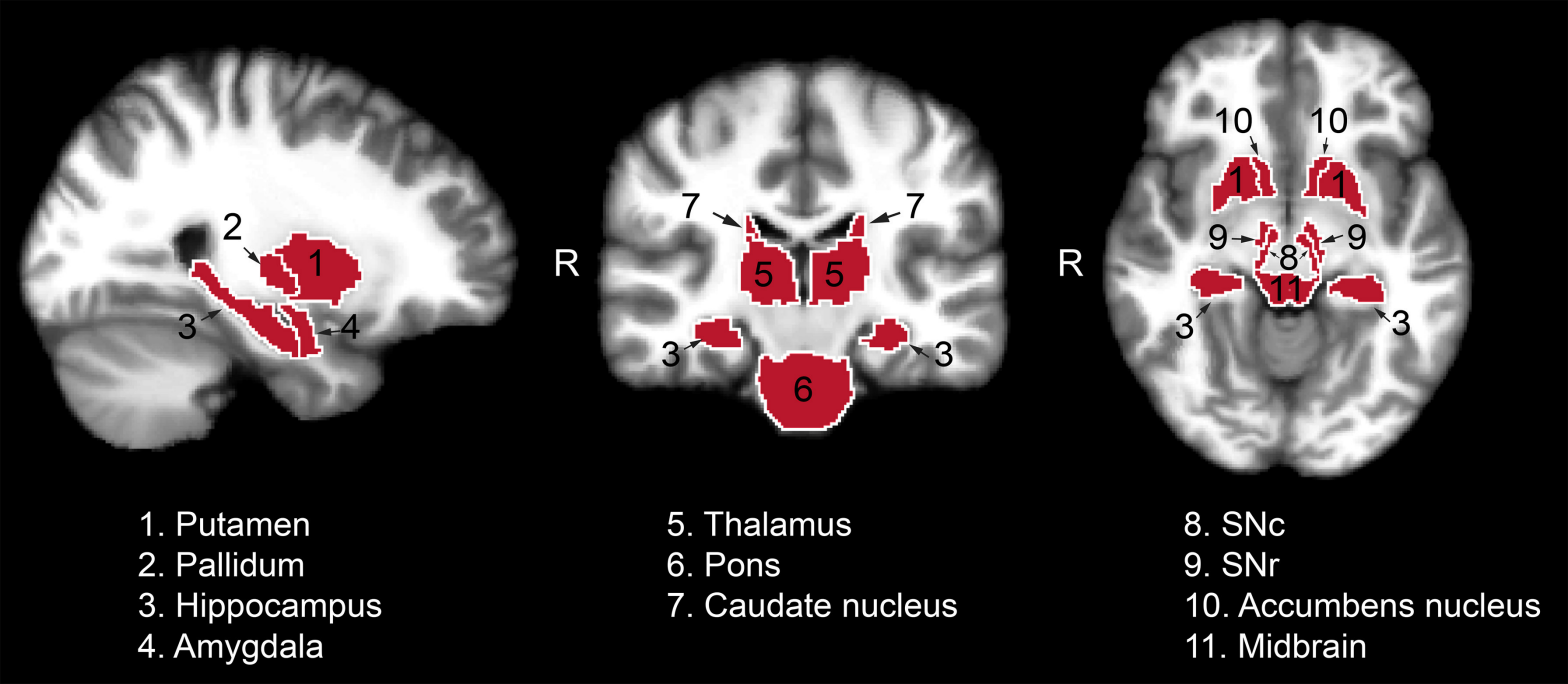
Regions of interest used for the analysis of the relaxation, DTI and functional data from one representative control subject. Masks identifying the regions of interest were transferred to standard space for visualization purposes. SNc, substantia nigra pars compacta; SNr, substantia nigra pars reticulata.

**Figure 2:**
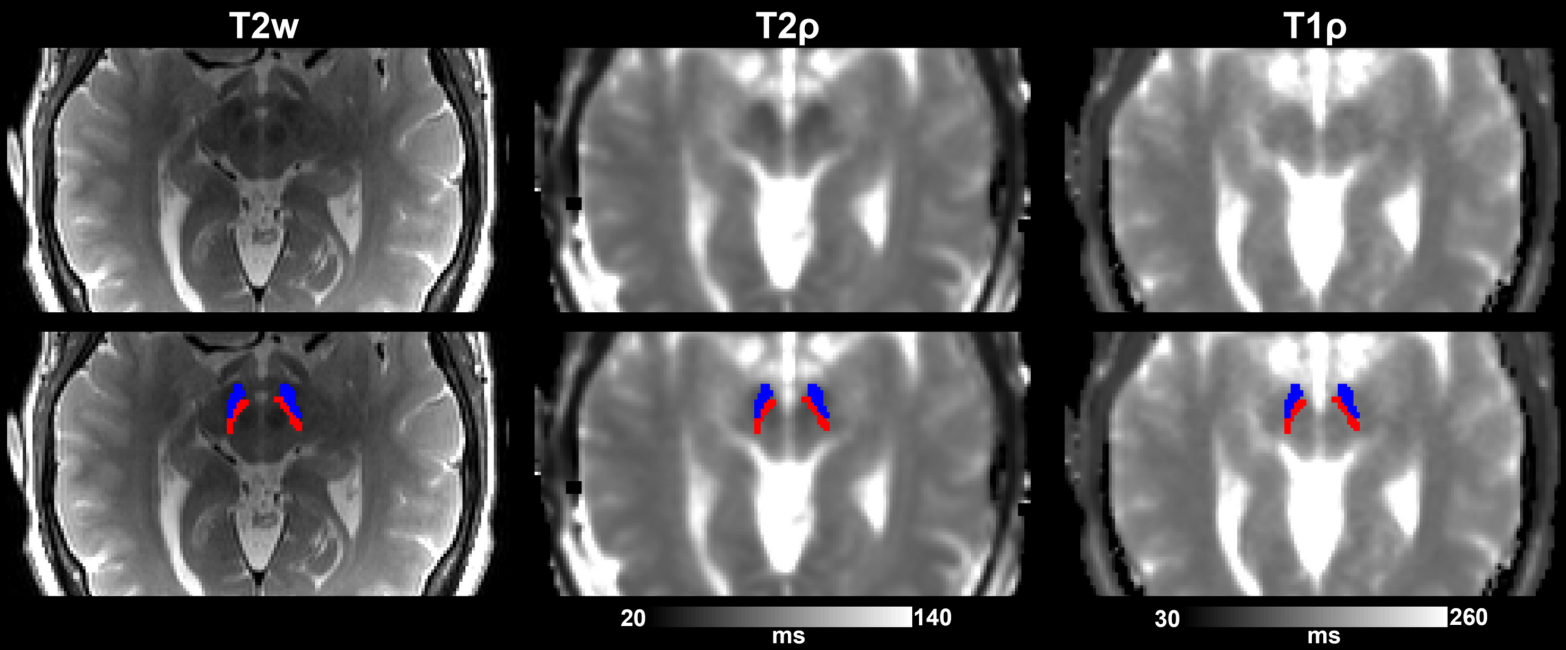
Substantia nigra ROIs (SNr, in blue, and SNc, in red) overlaid on T2-w images and adiabatic T_1ρ_, and T_2ρ_ maps from one representative control subject. Images are shown either without **(top row)** or with **(bottom row)** the overlay of the ROIs.

The following region of interest (ROI) masks were derived from FS automatic labeling of each subject's brain anatomy: caudate nucleus, pallidum, putamen, accumbens, amygdala, thalamus, hippocampus, and brainstem. With the exception of the brainstem, separate left and right hemisphere ROIs were generated for each structure. The brainstem was then manually separated to midbrain and pons regions. Additionally, left and right ROIs encompassing the substantia nigra reticulata (SNr), and separately the substantia nigra compacta (SNc), were manually drawn on each subject's T_2_-weighted image using itkSNAP software v.3.2.0 (Yushkevich et al., 2006) following the methodology described in our previous paper (Nestrasil et al., 2010). Representative examples of ROI selections are shown in Figures 1, 2.

Rigid-body motion correction was performed on adiabatic T_1ρ_, T_2ρ_ and RAFF4 measurements using the MCFLIRT algorithm in FSL (Jenkinson et al., 2012) with default options (Jenkinson et al., 2002). For T_1ρ_ and T_2ρ_ measures, all acquired images were firstly co-registered to the first acquisition and then to the averaged volume. The same algorithm was also applied on the two separate decay and recovery RAFF4 datasets to minimize the effect of movement between the two acquisitions. Relaxation time constant maps were calculated with MATLAB (MathWorks, Natick, MA) using in-house written routines and the Aedes software package (http://aedes.uef.fi). In particular, we used a 2-parameters non-linear fitting for estimating M_0_ and relaxation time in T_1ρ_ and T_2ρ_ acquisitions, and a 4-parameters non-linear fitting for estimating M_0_, M_ss_ (steady state value of magnetization), -M_z_ (initial magnetization value measured from the negative hemisphere) and relaxation time for RAFF4 acquisitions. The FreeSurfer BB-register algorithm (Greve and Fischl, 2009) was utilized to align the first acquired image of T_1ρ_, T_2ρ_, and RAFF4 measurements to the FS-preprocessed T1-weighted images. The transformation matrix was then applied to the parametric maps utilizing trilinear interpolation. The registration accuracy was visually verified. The quality of BB-register initialization was additionally quantified for the adiabatic relaxation maps by using the quality assessment value provided by the algorithm.

DTI datasets with opposite phase encoding were utilized to reduce susceptibility artifacts and field inhomogeneities using FSL TOPUP (Andersson et al., 2003). After motion and eddy-current correction (Andersson and Sotiropoulos, 2016), data were skull-stripped (Smith, 2002) and BB-register was applied to co-register DTI data to the T1-weighted image (Greve and Fischl, 2009). The parameters utilized for BB-register matched those used for motion-correcting the relaxation measurements, except that the first volume of relaxation measures was replaced by the b0 image in order to calculate the transformation matrix for DTI. The accuracy of registration was verified visually. The DTIFIT tensor model in FSL (Jenkinson et al., 2012) was fit to the co-registered DTI data to generate fractional anisotropy (FA) and mean diffusivity (MD) maps.

**Figure 3:**
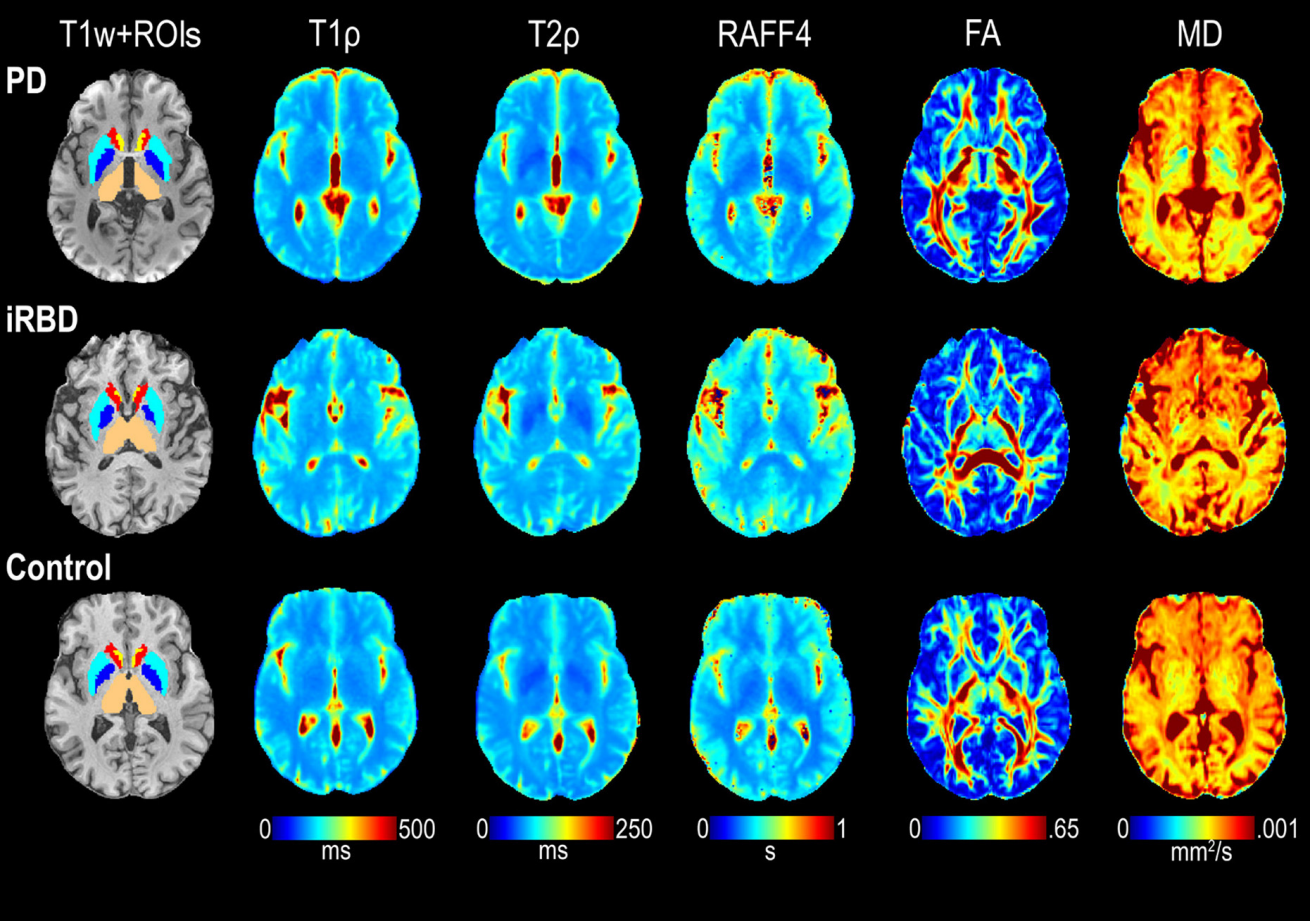
Representative maps of T_1ρ_, T_2ρ_, RAFF4, FA, and MD from one each of a control, iRBD and PD subject. ROIs of interest visible on the selected slices are also shown superimposed on the T1-weighted (T1w) images, including thalamus (beige), pallidum (dark blue), putamen (light blue), caudate (red), and accumbens (yellow).

T_1ρ_, T_2ρ_, RAFF4, FA, and MD values of co-registered images were averaged across voxels within each ROI for each subject. Where applicable, the masked MRI ROIs extracted from the left and right hemispheres were combined before averaging. Representative examples of relaxation and DTI maps are shown in Figure 3.

### Resting state functional connectivity

#### Pre-processing

Resting-state data were pre-processed and analyzed using AFNI (Cox, 1996), FS, and in-house routines based on Matlab R2013b. Data underwent the following pre-processing pipeline: (1) removal of first 10 volumes to allow for signal stabilization and for subjects to adapt to the new condition, (2) despiking (AFNI, 3dDespike), (3) slice-time correction (AFNI, 3dTshift), (4) realignment to the first EPI volume (AFNI, 3dvolreg), (5) coregistration of the first EPI volume to the FS-preprocessed T1-weighted image (FS, bbregister), and (6) normalization to 2 × 2 × 2 mm^3^ MNI space (FS, mri_vol2vol). To minimize data resampling, steps five and six were applied simultaneously, concatenating the two transformations (FS, mri_vol2vol). In addition, a general linear model was constructed to regress out baseline drift, motion and spurious variance from MNI normalized data (AFNI, 3dTproject). The following regressors were included in the model: a second order Legendre polynomial, a basis of sine and cosine to model frequencies outside the band 0.01–0.1 Hz, the six estimated motion parameters and their first derivative, the first five eigenvectors from the time series inside WM (after two voxels erosion; FS mask) and the first five eigenvectors from the time series inside CSF (after one voxel erosion; FS mask), following the aCompCor approach (Behzadi et al., 2007). To further reduce the impact of motion, data were censored by removing time points with more than 0.33 mm of displacement (estimated as the Euclidian norm of motion parameter derivatives) along with each previous time point. Censoring was applied during the regression step, removing time points from both data and regressors. The censoring threshold of 0.33 mm was selected so that at least 20 degrees of freedom (DOF) remained in the data. One PD subject could not meet this criterion for any reasonable choice of the censoring threshold and was therefore excluded from any further connectivity analysis, leaving a total of 10 controls, 8 iRBD and 8 PD subjects. The remaining DOF did not differ among groups (controls: 51.5 ± 7.9, RBD: 46.4 ±11.2, PD: 48.1 ± 7.8; ANOVA *p* = 0.48). Finally, conventional smoothing was not applied to the data since functional outcomes were not compared voxel-by-voxel but at the ROI level. However, since inter-subject variability in data smoothness, arising from different amounts of interpolation due to motion correction and/or spatial normalization, directly impacts local connectivity estimation, we set the effective data smoothness to an isotropic Gaussian FWHM of 4 mm (Maximo et al., 2013). This step was accomplished with the AFNI program 3dBlurToFWHM with the non-default option to avoid local smoothness estimation (i.e., whole-brain estimation only), in order to preserve the neuronal related smoothness.

#### Movement assessment

Framewise displacement (FD), as defined in Power et al. (2012), was computed to index the subject's movements during the resting-state scan. There was no significant difference in FD between controls and PD (controls: 0.15 ± 0.06 mm; PD: 0.18 ± 0.04; control vs. PD *p* = 0.10), however, iRBD subjects moved more than controls (iRBD: 0.22 ± 0.05; iRBD vs. control *p* = 0.004) and PD subjects (iRBD vs. PD *p* = 0.10). To partially take into account this issue we included FD as a nuisance covariate in all statistical tests of the functional metrics.

#### Metrics

Two functional metrics were calculated: seed-based functional connectivity (FC) and regional homogeneity (ReHo).

#### Seed-based FC

Time series within each of the ROIs described previously were averaged, yielding a single time series that served as seed. The correlation (Pearson's r) between the seed time series and the time series at each other voxel was then computed to quantify FC between the seed and the rest of the brain. To take into account the different number of DOF across subjects (due to the censoring procedure), correlation values were transformed in standard scores (Z) dividing the Fisher transformed *r*-value by the standard error of the Fisher's distribution:

(1) $$\begin{array}{l} Z=arctanh\left(r\right)*\sqrt{DOF-3}. \end{array}$$

For each subject and ROI, the seed-based FC was averaged within the functional network identified in the healthy control group. Specifically, the functional network was defined by a one-sample, one-tail, *t*-test on the seed-based FC maps of the healthy controls (*p* < 0.0005, minimum cluster size 20). We refer to this aggregate value of seed-based FC as network strength.

#### ReHo

Regional homogeneity was implemented as a metric to assess local connectivity (Zang et al., 2004). For each voxel in the brain, ReHo was computed as the Kendall's coefficient of concordance among the voxel time series and the time series of its 18 nearest neighbors (AFNI, 3dReHo). Resulting brain maps were then transformed into z-scores by subtracting the whole-brain mean and dividing by the whole-brain standard deviation. Aggregate ReHo values were finally extracted by averaging across voxels in each ROI previously described.

### Statistical analyses

**Figure 4:**
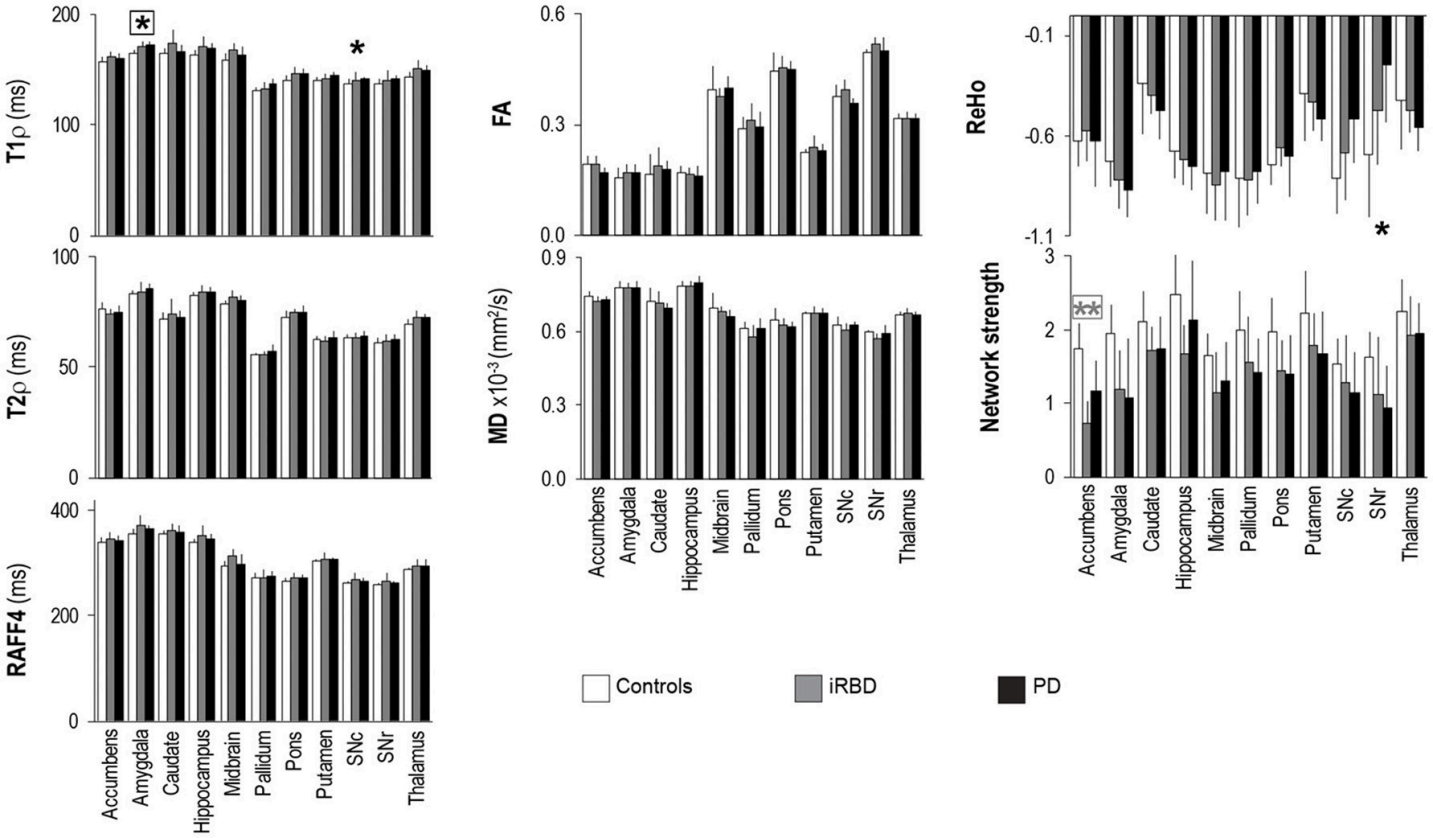
Group summaries of T_1ρ_, T_2ρ_, RAFF4, FA, MD, ReHo and network strength. *N* = 10, 8, 9 in the control, iRBD and PD groups, respectively, for T_1ρ_, T_2ρ_, and RAFF4, whereas *N* = 9, 8, 9 for FA and MD, and *N* = 10, 8, 8 for network strength and ReHo. Data shown as mean ± SD. ^*^ and ^**^ indicate, respectively, *p* < 0.05 and *p* < 0.005 with age-adjustments after Holm's correction (gray: iRBD vs. controls; black: PD vs. controls). Asterisks within a box indicate *p* < 0.05 after correcting FDR for multiple testing (7 modalities).

Subject age, clinical characteristics, and movement (FD) were summarized by group with means and standard deviations (SD) and compared using linear models that allowed for group-specific variances and provided Holm-corrected *p*-values for multiple comparisons among the groups (Holm, 1979); sex distribution was compared using Fisher's exact test. The average relaxation (T_1ρ_, T_2ρ_, and RAFF4), diffusion (FA, MD) and functional connectivity (network strength and ReHo) parameters were analyzed per ROI. For each of the 11 ROIs separately, each MR parameter was summarized by group (PD, iRBD, controls) using means and standard deviations, and compared across groups using linear models which allowed group-specific variances; we fit models with and without correction for age. Per ROI, type I error was controlled with Holm's adjustment for the 2 comparisons (PD vs. control and iRBD vs. control) and false discovery rate correction (Benjamini and Hochberg, 1995) was used for multiple testing of the 7 MRI outcomes. Results are presented both without and with the multiple-comparisons and the multiple-testing corrections (Figure 4, Supplementary Table 1).

## Results

Individuals enrolled in this study spanned from middle-age to older adults (Table 1). The age of PD and iRBD subjects was 66 years on average for both groups, while the healthy controls were on average 9 years younger (*p* < 0.02, corrected, for each of PD and iRBD vs. controls); the sex distribution was similar across all 3 groups. Disease duration spanned from 1 month to 4.5 years in the PD subjects, and from 1 to 15 years in the iRBD patients. As expected, PD subjects had significantly higher (worse) UPDRS scores and UPDRS-III scores than iRBD subjects (*p* = 0.00003 and *p* = 0.00009, respectively), but their MoCA values were comparable.

The adiabatic relaxation maps were well-registered to the anatomical images, with an average value of quality assessment of 0.21 ± 0.04 (normal range is between 0 and 1.1; 0 represents perfect registration initialization). The range of adiabatic T_1ρ_ (~130–180 ms), T_2ρ_ (~55–90 ms) and RAFF4 (~ 240–410 ms) measured at 3 T from the gray matter structures of interest were in good agreement with previous studies conducted at 4 T (Michaeli et al., 2007; Nestrasil et al., 2010; Liimatainen et al., 2015). In addition, rotating frame relaxation maps were highly reproducible among subjects. Indeed, between-subject variations among healthy controls were 3% for adiabatic T_1ρ_, T_2ρ_, and RAFF4 from the regions of interest. Diffusion parameters were more variable than relaxation parameters, with between-subjects variations of 9 and 4% for FA and MD, respectively. Between-subjects variations were considerably higher for functional connectivity parameters, namely 22 and 35% for network strength and ReHo, respectively.

Rotating frame relaxation parameters tended to be higher in patients than in controls for most of the ROIs (Figure 4, Supplementary Table 1). When age was not used as a covariate, significant Holm-corrected group-differences between PD patients and controls were observed for T_1ρ_ in the amygdala (*p* = 0.0001), hippocampus (*p* = 0.03), pons (*p* = 0.011), putamen (*p* = 0.014), substantia nigra compacta (*p* = 0.018), and thalamus (*p* = 0.013), while between iRBD and controls T_1ρ_ differences occurred in the amygdala (*p* = 0.012), hippocampus (*p* = 0.038), midbrain (*p* = 0.016), pons (*p* = 0.011), and thalamus (*p* = 0.013). Group differences between PD and controls were also observed for T_2ρ_ in the amygdala (*p* = 0.033) and thalamus (*p* = 0.08), while none of the RAFF4 findings were significant. Holm-corrected group differences between iRBD and controls occurred for T_2ρ_ and RAFF4 in the midbrain (*p* = 0.024 and *p* = 0.005, respectively). On the other hand, diffusion parameters exhibited only one Holm-corrected group difference, namely MD in SNr of iRB vs. controls (*p* = 0.001). Network strengths were smaller in patients than in controls for most regions. Holm-corrected group differences were significant for the network of the accumbens of both patient groups vs. controls (*p* < 0.013), and for the networks of the amygdala and SNr of PD vs. controls (*p* = 0.040 and *p* = 0.028, respectively). Finally, differences in ReHo were observed only between PD and controls in the SNc (*p* = 0.021) and SNr (*p* = 0.007).

Including age as a covariate demonstrated that most of the above mentioned differences were largely accounted for by age (Figure 4, Supplementary Table 1), especially for T_1ρ_ outcomes. A lengthening of T_1ρ_ in the amygdala of PD patients as compared to controls was however still robustly detected with Holm-correction after age-adjustment (*p* = 0.005). PD patients also maintained a significantly longer T_1ρ_ in the SNc (*p* = 0.046) as compared to controls. Between iRBD and controls, only trend-level group differences were observed after age-adjustments, namely for T_1ρ_ in the amygdala (*p* = 0.075), and for T_2ρ_ and RAFF4 in the midbrain (*p* = 0.08 and *p* = 0.064, respectively). Network strength was still lower in the accumbens of iRBD patients as compared to controls (*p* = 0.003), but only a trend was observed between PD and controls (*p* = 0.085). Moreover, age-adjustments and Holm-correction maintained a significantly lower ReHo in the SNr of PD vs. control (*p* = 0.035), while only trend level differences were reached for the amygdala (*p* = 0.067).

Finally, after additionally correcting FDR for multiple testing (7 modalities), the longer T_1ρ_ in the amygdala of PD vs. controls remained significant (*p* = 0.038), along with the smaller network strength of the accumbens of iRBD vs. controls (*p* = 0.018).

## Discussion

In the current study we recruited an iRBD group in addition to a mild-moderately affected PD group with the ultimate goal of identifying similarities in MRI signatures of both diseases. Both patient groups were selected to have no diagnosis of dementia, with MoCA scores not less than 25 (Table 1, and Supplementary Figure 1). In addition, while iRBD subjects had findings on their UPDRS-III evaluations (Table 1, and Supplementary Figure 1), they were not thought to meet a clinical diagnosis of PD (Hughes et al., 1992) at enrollment. Motivated by the group-differences previously observed between PD subjects and controls when using adiabatic T_1ρ_ and T_2ρ_ (Michaeli et al., 2007; Nestrasil et al., 2010; Tuite et al., 2012), here we piloted a more extended imaging protocol which included T_1ρ_ and T_2ρ_, RAFF4, DTI, and rsfMRI, and performed multi-slice acquisitions from the midbrain to characterize multiple subcortical brain regions rather than single slices of the brainstem as we used previously. Notably, mutli-slice mapping of T_1ρ_, T_2ρ_, and RAFF4 had never been implemented before, especially on a 3 Tesla clinical scanner. Here we demonstrate that 30-slices relaxation mapping is feasible with 5-min of acquisition time for each of T_1ρ_ and T_2ρ_ and 10-min for RAFF4. Thus the total acquisition time is similar to those employed for clinical MRI studies, and therefore such developments represent a critical step toward a future translation of the rotating frame methodologies to clinical trials. A multi-slice approach is needed for the purpose of obtaining more data that may allow an examination of regions involved in both motor and non-motor aspects of disease and that could be potentially useful in developing a prognostic biomarker (to predict whether iRBD patients are likely to develop PD) as well as a PD staging biomarker. With regard to the prognostic biomarker, based on clinical data, e.g., patients with greater cognitive impairment are more likely to have a different course than those without. If MRI methods can demonstrate pathology in sub-cortical regions involved in non-motor function, such as the amygdala or hippocampus, this information could be potentially useful in prognosis.

In principle, other MRI contrasts such as T_1_-Magnetization Transfer (MT) signal (so-called neuromelanin-sensitive scan Ogisu et al., 2013) and T_1_ relaxometry (Menke et al., 2009) could have been used, as they had been shown to reveal SN degeneration in PD. Such methods had been proven useful for visualizing and estimating the volumes of the SN structures. However, the neuromelanin-sensitive scan (Ogisu et al., 2013) is a qualitative approach based on signal intensity and it thus precludes a quantitative evaluation of SN tissue characteristics. On the other hand, T_1_-relaxometry is a quantitative method that allows quantification of tissue relaxation properties. However, the T_1_ measured in the SN did not reveal a difference between PD and controls (Menke et al., 2009), consistent with a lower sensitivity of free-precession relaxations to detect tissue degeneration as compared to rotating frame relaxations.

An important finding of the current study is that rotating frame relaxation parameters provided more robust characterizations of brain tissue as compared to DTI metrics, both in terms of observed coefficient of variations and in terms of their sensitivity to detect age-related and disease-related differences. The most prominent observation was that PD and iRBD groups share a lengthening of T_1ρ_ in a region that is non-motor related and appears abnormal during cognitive impairment and depression, i.e., the amygdala. However, group differences vs. controls were clearly more pronounced in PD patients than in iRBD patients, as they remained robustly significant for PD patients also after age-adjustments and corrections for multiple testing, whereas they reached only trend level in comparisons of iRBD subjects vs. controls. Dysfunction in olfactory, cardiovascular, sleep, sensory and cognitive functions is often present during PD, and even can precede the motor symptoms and the disease onset (for example, see Jellinger, 2015). Therefore, the differences between PD and iRBD vs. controls in non-motor areas detected by rotating frame T_1ρ_ MRI further support the critical importance of considering non-motor abnormalities in PD and iRBD. The T_1ρ_ results obtained in the amygdala of PD patients were overall consistent with previous findings of structural amygadalar deterioration in these patients (Bouchard et al., 2008; van Mierlo et al., 2015; Vriend et al., 2016). Since significant differences of amygadalar volumes were not observed in the present study (data not shown), and our PD subjects did not have significant mood or cognitive impairments, one may speculate that T_1ρ_ MRI may detect subtle tissue changes in the amygadala which occur prior to structural changes and/or symptom manifestations. Interestingly, the lengthening of T_1ρ_ in the amygdala as well as in SN regions of PD vs. controls, which is suggestive of neuronal degeneration in these areas, was accompanied by a deterioration of local functional connectivity as revealed by regional homogeneity.

Group differences were seen in rotating frame MRI parameters for other regions such as the thalamus, although such differences were largely explained by age. It is, however, still interesting to note that motor dysfunction in PD has been related to the change in activity of thalamic neurons in the motor circuits (Halliday, 2009). The slight lengthening of T_1ρ_ in the SNc of PD patients as compared to controls is in agreement with previous studies (Michaeli et al., 2007; Nestrasil et al., 2010), although the finding was not robust enough to survive after correcting FDR for multiple testing, most likely due to the limited number of subjects and/or due to lack of on-to-one age matching of PD and controls (although age was taken into account in the statistical analyses). Importantly, adiabatic T_2ρ_ was shown to pick up the difference in the SN of PD vs. control subjects in our previous studies conducted at 4 T (Michaeli et al., 2007; Nestrasil et al., 2010), but not in the current work. The differences in the outcomes among the current and previous studies could be attributed to the hardware used, i.e., higher magnetic field strength 4 T vs. 3 T (the lower field decreases sensitivity). Also, the multislice acquisition used in the current study may have impacted the efficiency of the contrast preparation module thus “diluting” the contrast as compared to the single slice used for the 4 T study. Importantly, in order to allow extended brain coverage, the spatial resolution used in this study was lower than previously used, thus leading to higher partial volume effects. Moreover, although the manual ROI segmentation of the SN was carefully performed, inaccuracies in placement are possible as there are unclear borders in T_2_-weighted images (Bolding et al., 2013). Differences between current and previous studies may also be due to different subject characteristics, with the most notable being a shorter disease duration of the current cohort (on average 2.5 years) as compared to our previous studies (on average 5 years in Michaeli et al., 2007; and 7 years in Nestrasil et al., 2010). Similar arguments apply for the pons, where T_1ρ_ differences were previously observed (Tuite et al., 2012). In fact, in the present cohort we observed lengthening of T_1ρ_ in the pons of PD vs. controls only when comparisons were not adjusted for age.

Finally, the long-distance connectivity as measured by network strength was significantly lower for the accumbens of iRBD subjects relative to controls, while such difference was not noted for the PD subjects. On the other hand, rotating frame relaxation and DTI parameters did not show evident abnormalities in the accumbens. Another putative signature of iRBD subjects was a longer RAFF4 and T_2ρ_ in the midbrain, that could again be related to changes in non-motor features as opposed to motor function in the iRBD group.

In future studies, it would be important to follow the subjects for at least 3 years to identify iRBD patients who remain stable, those who progressed to PD, and those who progressed to LBD. Changes in nigrostriatal circuits are expected mainly in iRBD converted to PD, and to a lessser extent in iRBD converted to LBD. At the same time, it will be critical to fully characterize the iRBD and PD subjects with extensive behavioral and cognitive tests in order to fully understand the implications of the MRI findings. In addition, the lack in the present study of a complete age matching among controls and patients was a nuisance that had to be resolved with the inclusion of age-adjustments in the statistical analyses, and will have to be avoided in future investigations. Yet it allowed appreciating for the first time the interesting property that rotating frame relaxations may be exquisitely sensitive to age, a feature that may even be exploited in investigations that target aging itself. From an acquisition perspective, the rotating frame relaxation protocols also allow great deal of flexibility. Depending on the hypothesis to be tested, for instance if a focus on only hippocampus and amygdala are desired, one may design a specific rotating frame multi-slice acquisition protocol that achieves high spatial resolution with a reduced number of slices, while guaranteeing full coverage of the selected regions of interest. If acquisition time is limited, one may choose to collect only adiabatic T_1ρ_ and resting-state fMRI, and skip entirely DTI which did not reveal sufficient sensitivity to detect group differences in this pilot investigation. On the other hand, other developments are currently underway to further optimize the acquisition times along with the intrinsic contrasts of the readout scheme. One promising direction is to implement magnetization preparation modules within a 3D readout sequence with virtually zero-echo times sequence, such as SWeep Imaging with Fourier Transformation (SWIFT) (Idiyatullin et al., 2006). Initial steps in this directions of research have already been proven successful in animal studies (Zhang et al., 2016).

Assessment of PD and iRBD subjects through combined microstructural and functional approaches may be a platform for comprehensive analysis of ongoing neurodegeneration. In essence, this approach may allow for staging of degeneration. In the present study, several outcomes showed trend-level differences in multiple regions, indicating the need of larger cohorts in future studies. Therefore, further studies on large cohorts of patients including longitudinal investigations of how iRBD advance to PD are necessary to characterize the ability of MRI metrics to be used as biomarkers of progression of iRBD into PD or other neurodegenerative diseases.

## Conclusion

We piloted for the first time the multi-slice mapping of rotating frame adiabatic T_1ρ_ and T_2ρ_ and non-adiabatic RAFF4 on a 3 Tesla scanner for human brain studies, which here involved control subjects along with non-demented iRBD and PD subjects. Rotating frame relaxations provided greater sensitivity to detect tissue abnormalities compared to conventional diffusion modalities that are commonly used to characterize tissue microstructure. The most prominent abnormality observed was a lengthening of T_1ρ_ in the amygdala of PD subjects, indicating neuronal degeneration in a non-motor area. This finding was even more prominent than what was detected by T_1ρ_ in the substantia nigra, i.e., the area related to the typical motor symptoms of PD. Significantly smaller network strength of the accumbens was also observed for iRBD subjects as compared to controls.

## Author contributions

SiM participated in design of the work, acquisition, analysis, interpretation of the data and preparing the manuscript. AS participated in analysis and interpretation of the data, and preparing the manuscript. DM participated in analysis and interpretation of the data, and preparing the manuscript. MN participated in acquisition and analysis of the data, and editing the manuscript. PCB participated in analysis and interpretation of the data, and editing the manuscript. PB participated in analysis and interpretation of the data, and editing the manuscript. EA participated in acquisition of the data, and editing the manuscript. FG participated in analysis and interpretation of the data, and editing the manuscript. LE participated in design of the work, analysis and interpretation of the data, and editing the manuscript. MH participated in design of the work, interpretation of the data, and editing the manuscript. IN participated in analysis and interpretation of the data, and editing the manuscript. PT participated in design of the work, interpretation of the data, and preparing the manuscript. ShM participated in design of the work, acquisition, interpretation of the data and preparing the manuscript.

### Conflict of interest statement

The authors declare that the research was conducted in the absence of any commercial or financial relationships that could be construed as a potential conflict of interest.
