# Corrigendum: Multi-modal Brain MRI in Subjects with PD and iRBD

**DOI:** 10.3389/fnins.2018.00446

**Published:** 2018-06-26

**Authors:** Silvia Mangia, Alena Svatkova, Daniele Mascali, Mikko J. Nissi, Philip C. Burton, Petr Bednarik, Edward J. Auerbach, Federico Giove, Lynn E. Eberly, Michael J. Howell, Igor Nestrasil, Paul J. Tuite, Shalom Michaeli

**Affiliations:** ^1^Department of Radiology, Center for Magnetic Resonance Research (CMRR), University of Minnesota, Minneapolis, MN, United States; ^2^Department of Pediatrics, University of Minnesota, Minneapolis, MN, United States; ^3^Central European Institute of Technology (CEITEC), Masaryk University, Brno, Czechia; ^4^MARBILab, Centro Fermi - Museo Storico Della Fisica e Centro di Studi e Ricerche Enrico Fermi, Rome, Italy; ^5^Department of Applied Physics, University of Eastern Finland, Kuopio, Finland; ^6^Fondazione Santa Lucia IRCCS, Rome, Italy; ^7^Division of Biostatistics, University of Minnesota, Minneapolis, MN, United States; ^8^Department of Neurology, University of Minnesota, Minneapolis, MN, United States

**Keywords:** rotating frame MRI, Parkinson's disease, iRBD, functional connectivity, DTI

In the original paper there were errors in **Figure 4** and in the text at page 7. **Figure 4** erroneously reported four asterisks, namely on top of T1ρ of putamen, T2ρ of midbrain, RAFF4 of midbrain, and ReHo of amygdala. The figure also did not report one asterisk on top of T1ρ of SNc. The correct version of **Figure 4** appears below.

**Figure 4:**
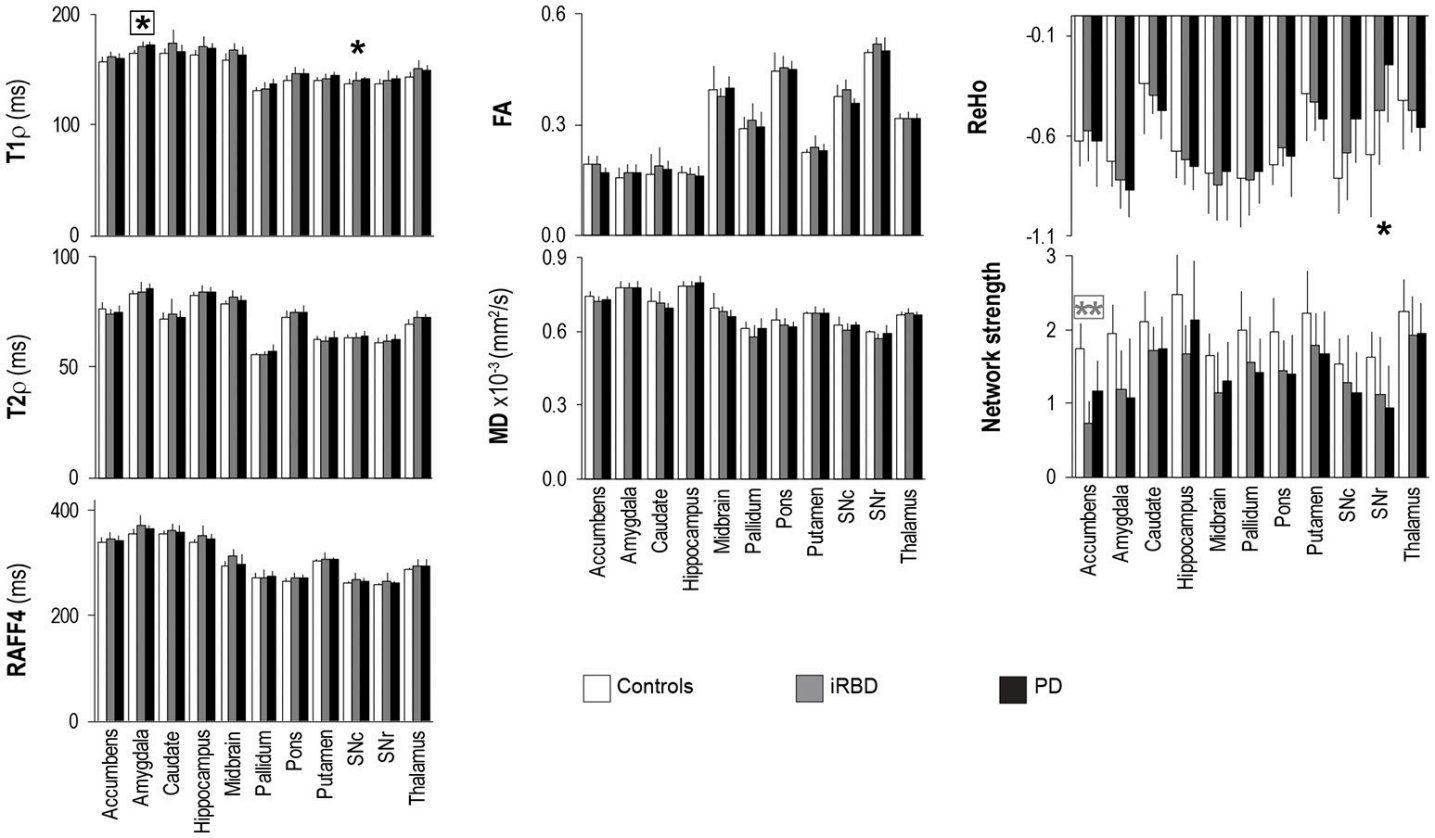
Group summaries of T_1ρ_, T_2ρ_, RAFF4, FA, MD, ReHo and network strength. *N* = 10, 8, 9 in the control, iRBD and PD groups, respectively, for T_1ρ_, T_2ρ_, and RAFF4, whereas *N* = 9, 8, 9 for FA and MD, and *N* = 10, 8, 8 for network strength and ReHo. Data shown as mean ± SD. ^*^ and ^**^ indicate, respectively, *p* < 0.05 and *p* < 0.005 with age-adjustments after Holm's correction (gray: iRBD vs. controls; black: PD vs. controls). Asterisks within a box indicate *p* < 0.05 after correcting FDR for multiple testing (7 modalities).

In addition, the text at page 7 “Group differences between PD and controls were also observed for T_2ρ_ in the amygdala (*p* = 0.033) and thalamus (*p* = 0.08)” should read as “Group differences between PD and controls were also observed for T_2ρ_ in the amygdala (*p* = 0.033) and thalamus (*p* = 0.008)”.

The authors sincerely apologize for these errors that may cause confusion for the reader. These errors however do not change the interpretation of the results and the main message of the study in any way, because the interpretation was based on the correct results reported in the Supplementary Table.

The original article has been updated.

## Conflict of interest statement

The authors declare that the research was conducted in the absence of any commercial or financial relationships that could be construed as a potential conflict of interest.

