# Supplementary material for: Multi-modal Brain MRI in Subjects with PD and iRBD
Source: Front Neurosci. 2017 Dec 19;11:709. doi: 10.3389/fnins.2017.00709 (PMC5742124; doi:10.3389/fnins.2017.00709)
Supplement: Supplementary file 1 [file Table1.docx]

| **Supplementary Table: Summary of group analyses.** Comparisons are shown with and without age correction. P-values are shown uncorrected (uncorr), and with progressive levels of multiple comparison corrections to account for the two comparisons iRBD vs controls and PD vs controls by Holm adjustments (Holm) and for the 7 MRI modalities by false discovery rate (FDR). Network strength and regional homogeneity (ReHo) are expressed in terms of z-scores. Details and references are in the methods section. N = 10, 8, 9 in the control, iRBD and PD groups, respectively, for T1ρ, T2ρ and RAFF4, whereas N = 9, 8, 9 for FA and MD, and N = 10, 8, 8 for network strength and ReHo. Highlighted cells indicate p < 0.05. | | | | | | | | | | | | | | | | | | | | | | |
| --- | --- | --- | --- | --- | --- | --- | --- | --- | --- | --- | --- | --- | --- | --- | --- | --- | --- | --- | --- | --- | --- | --- |
|  |  | **Group values** | | | | | | | | | **Comparisons without age correction** | | | | | | **Comparisons with age correction** | | | | | |
|  |  | **mean ± SD** | | | | | | | | | **p-values iRBD vs. controls** | | | **p-values PD vs. controls** | | | **p-values iRBD vs. controls** | | | **p-values PD vs. controls** | | |
| **modality** | **region** | **Control** | | | **iRBD** | | | **PD** | | | **uncorr** | **Holm** | **FDR** | **uncorr** | **Holm** | **FDR** | **uncorr** | **Holm** | **FDR** | **uncorr** | **Holm** | **FDR** |
| **T1ρ**  **(ms)** | **accumbens** | 158 | ± | 4 | 162 | ± | 4 | 160 | ± | 4 | 0.084 | 0.167 | 0.397 | 0.285 | 0.285 | 0.534 | 0.213 | 0.427 | 0.719 | 0.516 | 0.516 | 0.723 |
|  | **amygdala** | 165 | ± | 3 | 171 | ± | 5 | 172 | ± | 3 | 0.012 | 0.012 | 0.084 | 0.00003 | 0.0001 | 0.0004 | 0.075 | 0.075 | 0.525 | 0.003 | 0.005 | 0.038 |
|  | **caudate** | 164 | ± | 5 | 174 | ± | 13 | 167 | ± | 5 | 0.046 | 0.091 | 0.408 | 0.289 | 0.289 | 0.506 | 0.169 | 0.337 | 1.000 | 0.905 | 0.905 | 1.000 |
|  | **hippocampus** | 164 | ± | 4 | 171 | ± | 8 | 169 | ± | 5 | 0.038 | 0.038 | 0.252 | 0.015 | 0.030 | 0.172 | 0.219 | 0.438 | 0.937 | 0.275 | 0.438 | 0.937 |
|  | **midbrain** | 158 | ± | 6 | 167 | ± | 7 | 163 | ± | 8 | 0.008 | 0.016 | 0.055 | 0.179 | 0.179 | 0.628 | 0.062 | 0.124 | 0.289 | 0.464 | 0.464 | 1.000 |
|  | **pallidum** | 132 | ± | 3 | 133 | ± | 5 | 137 | ± | 6 | 0.466 | 0.466 | 0.727 | 0.026 | 0.053 | 0.314 | 0.991 | 0.991 | 1.000 | 0.184 | 0.368 | 0.879 |
|  | **pons** | 140 | ± | 5 | 147 | ± | 5 | 146 | ± | 4 | 0.010 | 0.011 | 0.078 | 0.006 | 0.011 | 0.078 | 0.119 | 0.238 | 0.950 | 0.131 | 0.238 | 0.805 |
|  | **putamen** | 140 | ± | 3 | 142 | ± | 4 | 144 | ± | 3 | 0.251 | 0.251 | 0.651 | 0.007 | 0.014 | 0.100 | 0.946 | 0.946 | 0.946 | 0.247 | 0.494 | 0.864 |
|  | **SNc** | 137 | ± | 4 | 140 | ± | 7 | 141 | ± | 2 | 0.275 | 0.275 | 0.493 | 0.009 | 0.018 | 0.073 | 0.302 | 0.302 | 0.843 | 0.023 | 0.046 | 0.322 |
|  | **SNr** | 137 | ± | 5 | 140 | ± | 10 | 141 | ± | 3 | 0.525 | 0.525 | 0.612 | 0.033 | 0.065 | 0.145 | 0.894 | 0.894 | 1.000 | 0.332 | 0.663 | 0.877 |
|  | **thalamus** | 143 | ± | 4 | 151 | ± | 7 | 150 | ± | 5 | 0.010 | 0.013 | 0.093 | 0.007 | 0.013 | 0.046 | 0.217 | 0.433 | 1.000 | 0.488 | 0.488 | 1.000 |
| **T2ρ**  **(ms)** | **accumbens** | 76 | ± | 3 | 74 | ± | 2 | 75 | ± | 3 | 0.191 | 0.382 | 0.668 | 0.357 | 0.382 | 0.534 | 0.331 | 0.662 | 0.773 | 0.511 | 0.662 | 0.773 |
|  | **amygdala** | 84 | ± | 2 | 84 | ± | 4 | 86 | ± | 2 | 0.596 | 0.596 | 0.835 | 0.017 | 0.033 | 0.094 | 0.767 | 0.767 | 0.895 | 0.104 | 0.207 | 0.363 |
|  | **caudate** | 72 | ± | 3 | 74 | ± | 7 | 73 | ± | 3 | 0.315 | 0.629 | 0.725 | 0.384 | 0.629 | 0.629 | 0.251 | 0.501 | 1.000 | 0.289 | 0.501 | 1.000 |
|  | **hippocampus** | 82 | ± | 1 | 84 | ± | 3 | 84 | ± | 2 | 0.112 | 0.112 | 0.262 | 0.031 | 0.063 | 0.172 | 0.389 | 0.634 | 0.937 | 0.317 | 0.634 | 0.937 |
|  | **midbrain** | 79 | ± | 2 | 82 | ± | 3 | 80 | ± | 2 | 0.012 | 0.024 | 0.057 | 0.060 | 0.060 | 0.418 | 0.040 | 0.080 | 0.281 | 0.240 | 0.240 | 1.000 |
|  | **pallidum** | 55 | ± | 1 | 56 | ± | 2 | 57 | ± | 3 | 0.887 | 0.887 | 0.887 | 0.058 | 0.116 | 0.314 | 0.724 | 0.724 | 1.000 | 0.199 | 0.398 | 0.879 |
|  | **pons** | 73 | ± | 3 | 75 | ± | 2 | 75 | ± | 2 | 0.139 | 0.145 | 0.323 | 0.073 | 0.145 | 0.327 | 0.431 | 0.575 | 0.950 | 0.287 | 0.575 | 0.805 |
|  | **putamen** | 62 | ± | 2 | 62 | ± | 2 | 64 | ± | 3 | 0.970 | 0.970 | 1.000 | 0.177 | 0.354 | 0.496 | 0.676 | 0.846 | 0.946 | 0.423 | 0.846 | 0.866 |
|  | **SNc** | 63 | ± | 1 | 63 | ± | 3 | 64 | ± | 2 | 0.863 | 0.863 | 0.863 | 0.251 | 0.501 | 0.585 | 0.999 | 0.999 | 0.999 | 0.429 | 0.857 | 0.879 |
|  | **SNr** | 61 | ± | 3 | 62 | ± | 3 | 63 | ± | 2 | 0.780 | 0.780 | 0.780 | 0.244 | 0.488 | 0.580 | 0.939 | 1.000 | 1.000 | 0.573 | 1.000 | 1.000 |
|  | **thalamus** | 70 | ± | 2 | 72 | ± | 3 | 72 | ± | 2 | 0.061 | 0.061 | 0.172 | 0.004 | 0.008 | 0.046 | 0.367 | 0.489 | 1.000 | 0.245 | 0.489 | 1.000 |
| **RAFF4 (ms)** | **accumbens** | 339 | ± | 11 | 346 | ± | 13 | 342 | ± | 10 | 0.277 | 0.555 | 0.776 | 0.613 | 0.613 | 0.716 | 0.172 | 0.344 | 0.719 | 0.345 | 0.345 | 0.603 |
|  | **amygdala** | 356 | ± | 8 | 370 | ± | 20 | 364 | ± | 9 | 0.064 | 0.101 | 0.176 | 0.050 | 0.101 | 0.141 | 0.150 | 0.300 | 0.618 | 0.368 | 0.368 | 0.429 |
|  | **caudate** | 355 | ± | 8 | 362 | ± | 12 | 359 | ± | 13 | 0.189 | 0.379 | 0.663 | 0.443 | 0.443 | 0.621 | 0.462 | 0.923 | 1.000 | 0.819 | 0.923 | 1.000 |
|  | **hippocampus** | 340 | ± | 7 | 354 | ± | 17 | 347 | ± | 9 | 0.036 | 0.072 | 0.252 | 0.074 | 0.074 | 0.172 | 0.132 | 0.263 | 0.937 | 0.553 | 0.553 | 0.937 |
|  | **midbrain** | 293 | ± | 11 | 313 | ± | 14 | 299 | ± | 18 | 0.003 | 0.005 | 0.036 | 0.389 | 0.389 | 0.681 | 0.032 | 0.064 | 0.281 | 0.797 | 0.797 | 1.000 |
|  | **pallidum** | 272 | ± | 8 | 274 | ± | 15 | 277 | ± | 8 | 0.793 | 0.793 | 0.887 | 0.231 | 0.463 | 0.716 | 0.703 | 0.703 | 1.000 | 0.244 | 0.488 | 0.879 |
|  | **pons** | 265 | ± | 8 | 272 | ± | 9 | 271 | ± | 9 | 0.094 | 0.189 | 0.323 | 0.107 | 0.189 | 0.327 | 0.285 | 0.570 | 0.950 | 0.401 | 0.570 | 0.805 |
|  | **putamen** | 303 | ± | 5 | 307 | ± | 13 | 306 | ± | 6 | 0.468 | 0.480 | 0.771 | 0.240 | 0.480 | 0.560 | 0.541 | 0.866 | 0.946 | 0.433 | 0.866 | 0.866 |
|  | **SNc** | 262 | ± | 5 | 268 | ± | 13 | 266 | ± | 5 | 0.221 | 0.221 | 0.493 | 0.046 | 0.092 | 0.214 | 0.350 | 0.602 | 0.843 | 0.301 | 0.602 | 0.879 |
|  | **SNr** | 258 | ± | 6 | 266 | ± | 16 | 263 | ± | 4 | 0.163 | 0.163 | 0.364 | 0.041 | 0.083 | 0.145 | 0.283 | 0.567 | 0.992 | 0.354 | 0.567 | 0.877 |
|  | **thalamus** | 288 | ± | 5 | 296 | ± | 10 | 296 | ± | 11 | 0.056 | 0.098 | 0.172 | 0.049 | 0.098 | 0.172 | 0.511 | 1.000 | 1.000 | 0.652 | 1.000 | 1.000 |
| **FA** | **accumbens** | 0.19 | ± | 0.04 | 0.19 | ± | 0.03 | 0.17 | ± | 0.02 | 0.979 | 0.979 | 1.000 | 0.167 | 0.334 | 0.534 | 0.514 | 0.514 | 0.719 | 0.052 | 0.104 | 0.362 |
|  | **amygdala** | 0.16 | ± | 0.01 | 0.17 | ± | 0.02 | 0.17 | ± | 0.02 | 0.043 | 0.086 | 0.176 | 0.047 | 0.086 | 0.141 | 0.082 | 0.165 | 0.577 | 0.105 | 0.165 | 0.363 |
|  | **caudate** | 0.17 | ± | 0.02 | 0.19 | ± | 0.02 | 0.18 | ± | 0.02 | 0.058 | 0.117 | 0.408 | 0.157 | 0.157 | 0.506 | 0.304 | 0.608 | 1.000 | 0.712 | 0.712 | 1.000 |
|  | **hippocampus** | 0.17 | ± | 0.03 | 0.16 | ± | 0.02 | 0.16 | ± | 0.01 | 0.753 | 1.000 | 1.000 | 0.565 | 1.000 | 1.000 | 0.559 | 0.803 | 0.937 | 0.402 | 0.803 | 0.937 |
|  | **midbrain** | 0.40 | ± | 0.08 | 0.38 | ± | 0.05 | 0.40 | ± | 0.07 | 0.520 | 1.000 | 1.000 | 0.895 | 1.000 | 1.000 | 0.390 | 0.781 | 0.990 | 0.868 | 0.868 | 1.000 |
|  | **pallidum** | 0.29 | ± | 0.02 | 0.31 | ± | 0.04 | 0.29 | ± | 0.03 | 0.100 | 0.200 | 0.700 | 0.614 | 0.614 | 0.716 | 0.393 | 0.786 | 1.000 | 0.674 | 0.786 | 1.000 |
|  | **pons** | 0.45 | ± | 0.01 | 0.45 | ± | 0.03 | 0.45 | ± | 0.02 | 0.395 | 0.790 | 0.790 | 0.576 | 0.790 | 0.790 | 0.679 | 0.679 | 0.950 | 0.256 | 0.512 | 0.805 |
|  | **putamen** | 0.23 | ± | 0.01 | 0.24 | ± | 0.02 | 0.23 | ± | 0.01 | 0.140 | 0.279 | 0.651 | 0.244 | 0.279 | 0.496 | 0.186 | 0.372 | 0.946 | 0.469 | 0.469 | 0.864 |
|  | **SNc** | 0.37 | ± | 0.02 | 0.39 | ± | 0.03 | 0.36 | ± | 0.03 | 0.132 | 0.264 | 0.493 | 0.154 | 0.264 | 0.462 | 0.368 | 0.368 | 0.843 | 0.117 | 0.233 | 0.545 |
|  | **SNr** | 0.50 | ± | 0.03 | 0.52 | ± | 0.03 | 0.50 | ± | 0.07 | 0.160 | 0.319 | 0.447 | 0.964 | 0.964 | 0.964 | 0.966 | 0.966 | 1.000 | 0.376 | 0.752 | 0.877 |
|  | **thalamus** | 0.32 | ± | 0.01 | 0.31 | ± | 0.01 | 0.32 | ± | 0.02 | 0.834 | 1.000 | 1.000 | 0.700 | 1.000 | 1.000 | 0.793 | 1.000 | 1.000 | 0.811 | 1.000 | 1.000 |
| **MD x10^-3^ (mm^2^/s)** | **accumbens** | 0.74 | ± | 0.02 | 0.72 | ± | 0.02 | 0.73 | ± | 0.01 | 0.085 | 0.170 | 0.397 | 0.106 | 0.170 | 0.534 | 0.164 | 0.328 | 0.719 | 0.251 | 0.328 | 0.603 |
|  | **amygdala** | 0.78 | ± | 0.03 | 0.78 | ± | 0.02 | 0.78 | ± | 0.02 | 0.904 | 1.000 | 1.000 | 0.820 | 1.000 | 1.000 | 1.000 | 1.000 | 1.000 | 0.929 | 1.000 | 1.000 |
|  | **caudate** | 0.72 | ± | 0.06 | 0.72 | ± | 0.05 | 0.69 | ± | 0.02 | 0.725 | 0.725 | 0.725 | 0.137 | 0.275 | 0.506 | 0.752 | 0.752 | 1.000 | 0.191 | 0.383 | 1.000 |
|  | **hippocampus** | 0.79 | ± | 0.02 | 0.78 | ± | 0.02 | 0.80 | ± | 0.03 | 0.639 | 0.639 | 0.759 | 0.257 | 0.514 | 0.767 | 0.740 | 0.740 | 0.937 | 0.320 | 0.640 | 0.937 |
|  | **midbrain** | 0.69 | ± | 0.06 | 0.68 | ± | 0.03 | 0.66 | ± | 0.03 | 0.553 | 0.553 | 0.870 | 0.173 | 0.346 | 0.681 | 0.586 | 0.586 | 0.990 | 0.210 | 0.420 | 1.000 |
|  | **pallidum** | 0.61 | ± | 0.03 | 0.58 | ± | 0.05 | 0.61 | ± | 0.04 | 0.097 | 0.195 | 0.700 | 0.979 | 0.979 | 0.979 | 0.206 | 0.413 | 1.000 | 0.857 | 0.857 | 1.000 |
|  | **pons** | 0.65 | ± | 0.05 | 0.63 | ± | 0.03 | 0.62 | ± | 0.02 | 0.230 | 0.230 | 0.323 | 0.104 | 0.207 | 0.327 | 0.277 | 0.295 | 0.950 | 0.147 | 0.295 | 0.805 |
|  | **putamen** | 0.67 | ± | 0.01 | 0.67 | ± | 0.03 | 0.68 | ± | 0.02 | 0.978 | 1.000 | 1.000 | 0.514 | 1.000 | 1.000 | 0.629 | 0.629 | 0.946 | 0.218 | 0.435 | 0.864 |
|  | **SNc** | 0.63 | ± | 0.03 | 0.61 | ± | 0.03 | 0.63 | ± | 0.01 | 0.176 | 0.352 | 0.493 | 0.917 | 0.917 | 0.917 | 0.154 | 0.308 | 0.843 | 0.879 | 0.879 | 0.879 |
|  | **SNr** | 0.60 | ± | 0.01 | 0.57 | ± | 0.02 | 0.59 | ± | 0.04 | 0.001 | 0.001 | 0.010 | 0.497 | 0.497 | 0.580 | 0.143 | 0.286 | 0.992 | 0.496 | 0.496 | 0.877 |
|  | **thalamus** | 0.67 | ± | 0.01 | 0.67 | ± | 0.02 | 0.67 | ± | 0.01 | 0.621 | 1.000 | 1.000 | 0.838 | 1.000 | 1.000 | 0.992 | 0.992 | 1.000 | 0.468 | 0.935 | 1.000 |
| **Network strength** | **accumbens** | 1.74 | ± | 0.36 | 0.73 | ± | 0.31 | 1.16 | ± | 0.41 | 0.000 | 0.001 | 0.004 | 0.012 | 0.012 | 0.084 | 0.001 | 0.003 | 0.018 | 0.085 | 0.085 | 0.362 |
|  | **amygdala** | 1.94 | ± | 0.40 | 1.20 | ± | 0.53 | 1.08 | ± | 0.79 | 0.052 | 0.052 | 0.176 | 0.020 | 0.040 | 0.094 | 0.286 | 0.363 | 0.618 | 0.182 | 0.363 | 0.429 |
|  | **caudate** | 2.12 | ± | 0.41 | 1.71 | ± | 0.33 | 1.74 | ± | 0.45 | 0.626 | 0.626 | 0.725 | 0.303 | 0.605 | 0.629 | 0.889 | 1.000 | 1.000 | 0.532 | 1.000 | 1.000 |
|  | **hippocampus** | 2.47 | ± | 0.55 | 1.67 | ± | 0.39 | 2.13 | ± | 0.80 | 0.156 | 0.311 | 0.545 | 0.658 | 0.658 | 0.767 | 0.192 | 0.383 | 0.937 | 0.686 | 0.686 | 0.937 |
|  | **midbrain** | 1.65 | ± | 0.30 | 1.15 | ± | 0.55 | 1.30 | ± | 0.54 | 0.311 | 0.622 | 0.870 | 0.315 | 0.622 | 0.870 | 0.428 | 0.849 | 0.990 | 0.424 | 0.849 | 1.000 |
|  | **pallidum** | 2.00 | ± | 0.53 | 1.56 | ± | 0.61 | 1.42 | ± | 0.48 | 0.435 | 0.435 | 0.727 | 0.067 | 0.134 | 0.314 | 0.770 | 0.770 | 1.000 | 0.251 | 0.502 | 0.879 |
|  | **pons** | 1.98 | ± | 0.46 | 1.44 | ± | 0.42 | 1.40 | ± | 0.54 | 0.561 | 0.561 | 0.655 | 0.117 | 0.233 | 0.327 | 0.990 | 0.990 | 0.990 | 0.445 | 0.889 | 0.997 |
|  | **putamen** | 2.23 | ± | 0.56 | 1.79 | ± | 0.44 | 1.68 | ± | 0.57 | 0.551 | 0.551 | 0.771 | 0.166 | 0.331 | 0.496 | 0.869 | 0.869 | 0.946 | 0.365 | 0.730 | 0.866 |
|  | **SNc** | 1.54 | ± | 0.34 | 1.27 | ± | 0.66 | 1.16 | ± | 0.53 | 0.747 | 0.747 | 0.863 | 0.190 | 0.379 | 0.531 | 0.860 | 0.860 | 0.999 | 0.334 | 0.669 | 0.879 |
|  | **SNr** | 1.62 | ± | 0.35 | 1.13 | ± | 0.78 | 0.93 | ± | 0.58 | 0.208 | 0.208 | 0.364 | 0.014 | 0.028 | 0.099 | 0.506 | 0.506 | 0.992 | 0.139 | 0.277 | 0.877 |
|  | **thalamus** | 2.25 | ± | 0.42 | 1.92 | ± | 0.54 | 1.95 | ± | 0.41 | 0.840 | 1.000 | 1.000 | 0.520 | 1.000 | 1.000 | 0.733 | 1.000 | 1.000 | 0.723 | 1.000 | 1.000 |
| **ReHo** | **accumbens** | -0.63 | ± | 0.13 | -0.58 | ± | 0.15 | -0.63 | ± | 0.23 | 0.508 | 1.000 | 1.000 | 0.946 | 1.000 | 1.000 | 0.954 | 1.000 | 1.000 | 0.686 | 1.000 | 1.000 |
|  | **amygdala** | -0.72 | ± | 0.13 | -0.82 | ± | 0.15 | -0.87 | ± | 0.14 | 0.759 | 0.759 | 0.886 | 0.097 | 0.193 | 0.225 | 0.441 | 0.441 | 0.618 | 0.033 | 0.067 | 0.234 |
|  | **caudate** | -0.34 | ± | 0.25 | -0.40 | ± | 0.09 | -0.47 | ± | 0.14 | 0.270 | 0.270 | 0.629 | 0.113 | 0.226 | 0.506 | 0.533 | 0.553 | 1.000 | 0.277 | 0.553 | 1.000 |
|  | **hippocampus** | -0.68 | ± | 0.13 | -0.72 | ± | 0.12 | -0.75 | ± | 0.12 | 0.651 | 0.651 | 0.759 | 0.286 | 0.571 | 0.767 | 0.920 | 1.000 | 1.000 | 0.560 | 1.000 | 1.000 |
|  | **midbrain** | -0.78 | ± | 0.21 | -0.85 | ± | 0.17 | -0.78 | ± | 0.24 | 0.582 | 1.000 | 1.000 | 0.966 | 1.000 | 1.000 | 0.986 | 1.000 | 1.000 | 0.568 | 1.000 | 1.000 |
|  | **pallidum** | -0.81 | ± | 0.24 | -0.82 | ± | 0.17 | -0.77 | ± | 0.16 | 0.259 | 0.519 | 0.727 | 0.312 | 0.519 | 0.716 | 0.548 | 1.000 | 1.000 | 0.692 | 1.000 | 1.000 |
|  | **pons** | -0.74 | ± | 0.10 | -0.66 | ± | 0.10 | -0.70 | ± | 0.20 | 0.097 | 0.194 | 0.323 | 0.516 | 0.516 | 0.602 | 0.433 | 0.866 | 0.990 | 0.997 | 0.997 | 0.997 |
|  | **putamen** | -0.39 | ± | 0.24 | -0.43 | ± | 0.14 | -0.52 | ± | 0.11 | 0.097 | 0.097 | 0.651 | 0.046 | 0.092 | 0.322 | 0.140 | 0.142 | 0.946 | 0.071 | 0.142 | 0.864 |
|  | **SNc** | -0.81 | ± | 0.18 | -0.68 | ± | 0.24 | -0.51 | ± | 0.22 | 0.346 | 0.346 | 0.493 | 0.010 | 0.021 | 0.073 | 0.543 | 0.543 | 0.843 | 0.051 | 0.101 | 0.355 |
|  | **SNr** | -0.69 | ± | 0.31 | -0.48 | ± | 0.27 | -0.24 | ± | 0.29 | 0.086 | 0.086 | 0.299 | 0.003 | 0.007 | 0.048 | 0.181 | 0.181 | 0.992 | 0.017 | 0.035 | 0.242 |
|  | **thalamus** | -0.42 | ± | 0.24 | -0.47 | ± | 0.11 | -0.56 | ± | 0.12 | 0.084 | 0.084 | 0.172 | 0.040 | 0.080 | 0.172 | 0.177 | 0.189 | 1.000 | 0.095 | 0.189 | 1.000 |
