# Supplementary material for: Multi-modal Brain MRI in Subjects with PD and iRBD
Source: Front Neurosci. 2017 Dec 19;11:709. doi: 10.3389/fnins.2017.00709 (PMC5742124; doi:10.3389/fnins.2017.00709)

Supplementary Figure. Distributions of MoCA scores and UPDRS part III subscale measured in the two groups: iRBD subjects (n=8) and PD subjects (n=9).

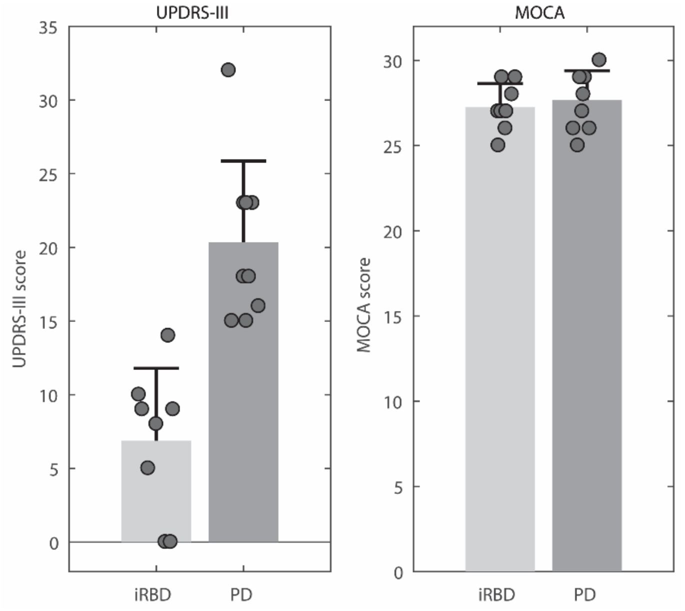

Supplement: Supplementary file 2 [file Image1.PDF]
